# Successful surgical management of primary hyperparathyroidism during pregnancy: a rare case report

**DOI:** 10.1097/MS9.0000000000000381

**Published:** 2023-03-25

**Authors:** Asil Musleh, Oadi N. Shrateh, Khaled Abbadi, Malvina Asbah, Abdellatif Khader

**Affiliations:** a Department of General Surgery, Palestinian Medical Complex (PMC), Ramallah; b Faculty of Medicine, Al-Quds University, Jerusalem, Palestine

**Keywords:** case report, gestational PHPT, hypercalcemia, pancreatitis, pregnancy

## Abstract

Primary hyperparathyroidism (PHPT) in pregnancy is a rare condition. Because of gestational physiologic changes, it is easily under-recognized; in some cases, patients may remain symptomless while maintaining high serum calcium levels, posing a risk to the health of both mother and fetus.

**Case Presentation:**

Our patient is a pregnant woman in her 30th week of gestation admitted to the hospital with typical features of acute pancreatitis. All possible causes of acute pancreatitis were ruled out. Further investigation, including neck ultrasound, revealed a hypoechoic, well-defined, heterogeneous, and vascularized lesion measuring 1.9×1.7 cm, seen posterior to the left thyroid lobe and mostly representing a parathyroid adenoma. The patient was diagnosed to have a PHPT as the etiologic factor and underwent a successful parathyroidectomy after the failure of medical treatment.

**Discussion and Conclusion:**

Pregnancy-related parathyroid disease is uncommon. Several changes in calcium-regulating hormones occur during pregnancy, making the diagnosis of PHPT noticeably challenging. Therefore, serum calcium levels must be closely monitored during pregnancy for optimization of maternal and fetal outcomes. For the same reason, the appropriate management of gestational PHPT is mandatory, either medically or surgically.

## Introduction

Highlights

- Hypercalcemia-induced acute pancreatitis due to primary hyperparathyroidism is a rare but serious and life-threatening medical condition during pregnancy.
- Gestational primary hyperparathyroidism is a rare condition that can be misdiagnosed; it poses a risk to the mother’s and fetus’s health.
- To optimize maternal and fetal outcomes, early diagnosis and medical and/or surgical management are mandatory.

Primary hyperparathyroidism (PHPT) is caused by an excessive abnormal production of parathyroid hormone (PTH) by one or more parathyroid glands^1^. It is the most common cause of hypercalcemia and the third most prevalent endocrine disease (after diabetes and thyroid disorders). It tends to affect 0.3% of the overall population, with women having a two times higher incidence^2^ and pregnant and nonpregnant women having a similar incidence^3^. PHPT is most commonly caused by sporadic parathyroid adenomas or carcinomas, but it can also be seen in accordance with multiple endocrine neoplasia, rare congenital syndromes, or metabolic disorders^1^. Gestational PHPT is a rare condition that can be misdiagnosed; it poses a risk to the mother’s and fetus’s health. Pregnancy-related physiological changes may mask the signs and symptoms of PHPT; hypoalbuminemia, placental transfer of calcium, and an elevated glomerular filtration rate all contribute to lower serum calcium levels. Simultaneously, estrogens inhibit PTH-mediated bone resorption, resulting in a dose-related decrease in serum calcium in pregnant patients^3^. In women, the most frequent clinical manifestations are renal calculi, excessive vomiting, pancreatitis, or hypercalcemic crisis. Even in 67% of patients, maternal complications have been reported^4^. Untreated disease can frequently jeopardize fetal development and cause death. Complications cannot be anticipated depending on the duration of the illness or calcium levels. To avoid complications, early recognition and medical and/or surgical management should be considered. We described a case of gestational PHPT presented with acute pancreatitis and underwent successful parathyroidectomy. This case report has been reported in line with the SCARE (Surgical CAse REport) Criteria^5^.

## Case presentation

**Figure 1:**
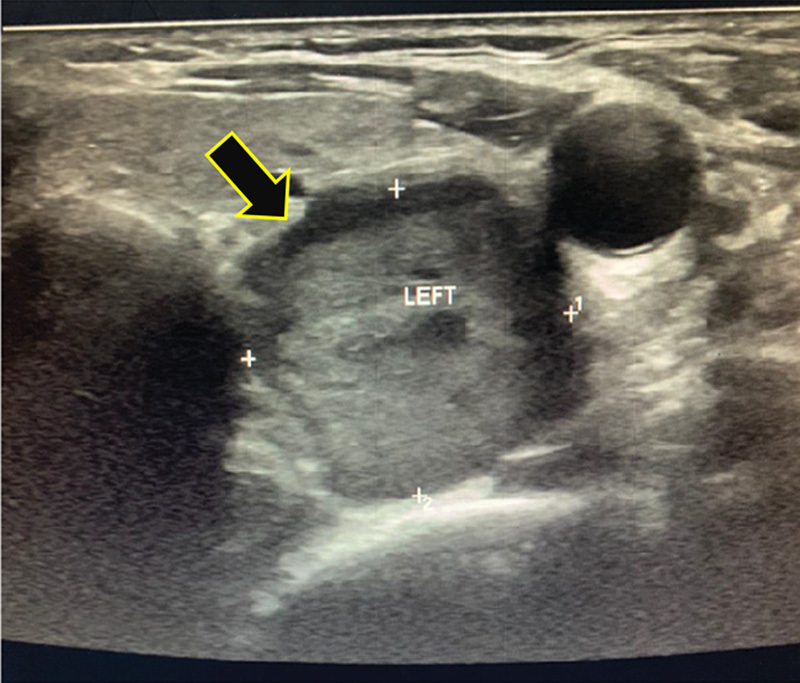
Neck ultrasound showing a hypoechoic, well-defined, heterogeneous, and vascularized lesion measuring 1.9×1.7 cm, seen posterior to the left thyroid lobe (arrow).

**Figure 2:**
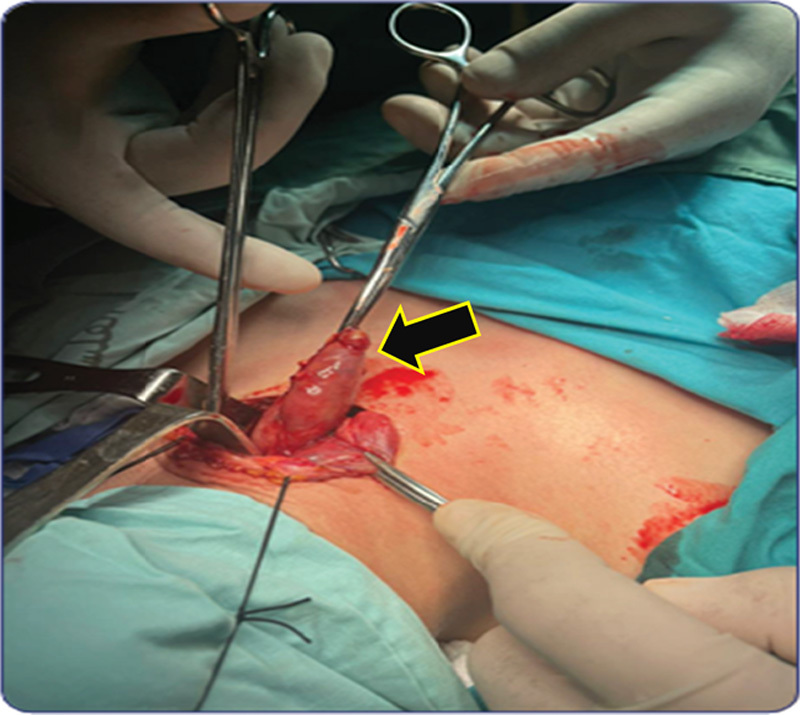
Intraoperative image showing resection of the parathyroid adenoma (arrow).

A 31-year-old female patient, gravida 4, para 3, presented at her 30th week of gestation for epigastric pain. The pain was gradual in onset, epigastric, progressive, and constant, radiating to the back with no relieving factors, of 2 days’ duration, and associated with nausea and vomiting once of normal gastric content. She denies any fever, previous similar attacks, or any other complaint. The patient reported no personal and/or family history of cancer; any acute, repeat, or discontinued medications; any allergies; or any genetic or psychosocial issues. Past medical and surgical histories were free. On physical examination, the patient was fully conscious, alert, and oriented, with stable vital signs, a gravid abdomen, soft laxity with severe epigastric tenderness, and voluntary guarding. Laboratory tests showed leukocytosis with a left shift (12 800/μl with 88% neutrophils), alanine aminotransferase of 13 U/l, aspartate aminotransferase of 27 U/l, alkaline phosphatase of 313 IU/l, direct bilirubin of 0.3 mg/dl, total bilirubin of 0.9 mg/dl, and 10 times elevated amylase of 1383 IU/l. Pancreatitis was diagnosed based on the clinical presentation and laboratory findings. The patient was kept NPO (nil per os, meaning nothing by mouth) with pain control medications and intravenous (i.v.) fluids. While investigating the etiology of pancreatitis, an ultrasound of her abdomen showed an enlarged liver with a 19 cm span at mid-clavicular, regular contour, normal echotexture, and no dilatation of intrahepatic biliary ducts. Gallbladder is normally distended, with no stones or thickening of the walls. Common bile duct is not dilated. The visualized proximal parts of the pancreas appear edematous and echogenic and measure 3.5 cm. However, her calcium level was found to be significantly elevated, with a value of 11.7 mg/dl with low serum phosphorus and magnesium levels of 1.2 and 1.16 mg/dl, respectively, and a serum albumin level of 2.5 g/dl. PTH levels were elevated at 701 pg/ml and 25-hydroxyvitamin D levels were 21 ng/ml. Based on the above-mentioned results, PHPT was identified, and the resulting hypercalcemia was thought to be the underlying pathologic agent of her acute pancreatitis. A neck ultrasound revealed a well-defined heterogeneous, mostly hypoechoic, solidly vascularized lesion measuring 1.9×1.7 cm, seen posterior to the left thyroid lobe and mostly representing a parathyroid adenoma. The right thyroid lobe shows a well-defined spongiform nodule measuring 1×0.6 cm and a tiny pure cyst; otherwise, the left thyroid lobe is normal in size and echotexture, with no enlarged cervical lymph nodes (Fig. 1). At the same time, the initial target to reduce the patient’s serum calcium level failed despite various attempts using aggressive saline infusion. Moreover, the patient refused all other medical therapies due to their potential adverse effects on pregnancy, despite discussing their risks and benefits with herself and her family. Thus, a multidisciplinary decision involving the gynecology, endocrinology, internal medicine, and surgery teams was made, and the patient was advised to have an emergency parathyroidectomy. So, parathyroidectomy was performed after 7 days of diagnosis, with a subsequent significant intraoperative drop in PTH (from 1331 to 234 to 18 pg/ml) indicating adequate adenoma removal (Fig. 2). The procedure was performed by a consultant at the general surgery department of a government hospital. The patient was followed up for 3 weeks without any reported complications, adverse events, or readmissions. The pregnancy was continued without any maternal and/or fetal complications.

## Follow-up and outcomes

The patient experienced a rapid resolution of her acute pancreatitis with improvement in her abdominal pain. On postoperative day 1, the calcium level dropped to 9.2 mg/dl, the phosphorus level was 1.3 mg/dl, and the magnesium level was 1.6 mg/dl. Simultaneously, the patient complained of perioral numbness for a few days, with a positive Chvostek sign and serum calcium of 7.9 mg/dl; oral calcium was started and gradually increased to 1200 mg three times daily, and calcitriol 0.5 μg was started. On the 10th postparathyroidectomy day, the patient was successfully discharged home, and a 3-week follow-up revealed no symptoms with a calcium level of 8.6 mg/dl and a PTH level of 110 pg/ml. Calcium and calcitriol replacement were therefore discontinued.

## Discussion

We discuss a rare condition occurring during pregnancy. Even though it is uncommon, it is crucial to include PHPT in the differential diagnosis of acute pancreatitis during pregnancy.

PHPT is a common endocrine disease with an estimated incidence of 0.3% in the general population, a twice higher incidence in women, and 8 cases per 100 000 people per year in women of childbearing age^6^,^7^. It is the third most common endocrine disease in pregnancy, affecting less than 1% of all pregnant women. Primary adenoma is the most common etiology of PHPT^8^. Acute pancreatitis is rare in pregnancy, occurring in ∼3 out of every 10 000 pregnancies^9^. Among the various etiological factors of acute pancreatitis, the most common causes in pregnancy are gallstones (66%), idiopathy (17%), alcohol abuse (12%), hyperlipidemia (4%), and less commonly, hyperparathyroidism, trauma, medication, and fatty liver of pregnancy^10^. Hypercalcemia due to PHPT has a variety of consequences and a spectrum of complications for the pregnant woman and her fetus, and there seems to be a strong positive correlation between calcium concentrations and pregnancy complications, with the risk being particularly increased if total calcium levels are greater than 2.85 mmol/l (>11.42 mg/dl)^11^, ranging from mild, vague symptoms to life-threatening complications^12^. PHPT causing acute pancreatitis in pregnancy is a morbid medical condition with a mortality rate of less than 1% for the mother and 5% for the fetus and has a spectrum of maternal and fetal complications^6^,^7^,^12^.

A systemic review included 75 manuscripts that described 382 cases of hyperparathyroidism during pregnancy. The average maternal age was 31 years old. In total, 108 cases (28.3%) had parathyroidectomy while pregnant, while 274 cases (71.7%) were treated nonsurgically. The majority of surgeries (67.6%) were performed during the second trimester. Complications and/or deaths were less likely to occur after second-trimester surgery (4.48%) compared to third-trimester surgery (21.1%). Despite the fact that nine surgically treated cases resulted in infant complications and/or death, none of these nine cases had any surgical complications. Despite these complications, the overall infant complication rate for surgical patients remained lower than that of conservative therapy patients (9.1% versus 38.9%)^13^.

The diagnosis of PHPT in pregnancy is rare and challenging since most cases are mild and asymptomatic (80% of cases). With the exception of normal physiological changes in pregnancy (plasma volume expansion that results in low total serum calcium; the upper limit of normal total calcium in pregnancy is considered 9.5 mg/dl due to decreased albumin levels, facilitated transport of calcium across the placenta, and increased glomerular filtration rate), the diagnosis is similar to nonpregnant patients. Alternatively, ionized calcium does not change significantly with pregnancy and may represent a better assay to assess serum calcium levels during pregnancy^12^,^14^,^15^.

The biochemical diagnosis of PHPT is based on elevated serum albumin-adjusted calcium concentrations or elevated serum ionized calcium concentrations with an inappropriately normal or elevated PTH concentration^16^. Once PHPT is diagnosed biochemically, imaging should follow to determine the etiology. Though it is reasonable to aim for two different preoperative imaging methods. Neck ultrasound is usually the first modality used to identify the parathyroid adenoma (sensitivity 69%, specificity 94%) in combination with either a 99mTc-MIBI scan or a four-dimensional dynamic contrast-enhanced MRI, as nuclear scans are contraindicated in pregnancy due to the fetal risk from ionizing radiation. However, other imaging methods or ultrasonography without additional imaging may be considered for selected cases, taking into account the local expertise and the individual risks and benefits^11^,^14^.

International guidelines recommend parathyroidectomy in patients below the age of 50 years; usually conservative treatment options including oral and i.v. rehydration and cinacalcet should be temporary until surgery. Cinacalcet has been used in several pregnant women without significant safety concerns, but safety evidence are still considered insufficient for official approval. Safety concerns argue against the use of bisphosphonates regarding its effects on fetal bone development. Subcutaneous calcitonin does not cross the placenta and can help reduce maternal calcium levels, but its benefits are often short-lived because tachyphylaxis soon develops^11^,^16^–^18^. Studies showed that women with gestational PHPT who underwent parathyroidectomy during pregnancy reported a significantly lower infant complication rate for surgery versus medical therapy (9.1% versus 38.9%)^11^. The timing of surgery is recommended in the second trimester, especially if albumin-adjusted calcium is above 2.85 mmol/l (>11.42 mg/dl) and/or above 0.25 mmol/l (>1 mg/dl) upper limit of normal, and/or ionized calcium is above 1.45 mmol/l (>5.81 mg/dl), as complications are higher in the first and third trimesters. However, maternal–fetal complications or failure of conservative medical therapy warrant urgent parathyroidectomy regardless of fetal gestational age^11^,^16^.

## Conclusion

In summary, hypercalcemia due to PHPT is a rare but serious and life-threatening medical condition that is likely underdiagnosed in pregnancy, in which prompt diagnosis and aggressive treatment are important to prevent maternal, fetal, and neonatal consequences. Surgical treatment is associated with better outcomes compared to conservative management. In this case study, we affirm the significance of early detection and management of PHPT during pregnancy.

## Ethical approval

Our institution has exempted this study from ethical review.

## Patient consent

Written informed consent was obtained from the patient for the publication of this case report. A copy of the informed consent is available for review by the Editor-in-Chief of this journal on request.

## Sources of funding

This research did not receive any specific grant from funding agencies in the public, commercial, or not-for-profit sectors.

## Author contribution

A.M., M.A., K.A., and O.N.S.: writing the manuscript; A.K.: imaging description and reviewing and editing the manuscript.

## Conflicts of interest disclosure

The authors declare that they have no known competing financial interests or personal relationships that could have appeared to influence the work reported in this paper.

## Research registration unique identifying number (UIN)

1. None.

## Guarantor

Oadi N. Shrateh.

## Provenance and peer review

Not commissioned, externally peer-reviewed.

## Authorship

All authors attest that they meet the current ICMJE criteria for authorship.
